# Novel gene rearrangement in the complete mitochondrial genome of *Telenomus remus* (Hymenoptera: Scelionidae)

**DOI:** 10.1080/23802359.2021.1915210

**Published:** 2021-11-18

**Authors:** Weiwei Li, Yunfei Wu, Bin Chen

**Affiliations:** a State Key Laboratory for Conservation and Utilization of Bio-Resources in Yunnan, Yunnan Agricultural University, Kunming, China; b Kunming Institute of Zoology, Chinese Academy of Sciences, Kunming, China; c Department of Entomology, College of Pant Protection, China Agricultural University, Beijing, China

**Keywords:** Mitochondrial genome, gene rearrangement, parasitic wasps, *Telenomus remus*

## Abstract

The current study describes the complete mitochondrial genome (mitogenome) of an egg parasitoid wasp, *Telenomus remus* Nixon 1937. This mitogenome is 16,014 bp in length, consisting of 37 typical coding genes (13 protein-coding genes, 22 transfer RNA genes and two ribosomal RNA genes). The start codons of the protein-coding genes are ATN and the stop codons are TAA or TAG. The secondary structures of most transfer RNA genes could be detected, except for *trnS(AGN)* and *trnQ*. Rearrangements of 14 transfer RNA genes in the mitogenome has generated a novel gene order, including two new gene clusters, *trnN*-*trnF*-*trnS(AGN)*-*trnR* between *ND3* and *ND5*, and *trnM*-*trnV*-CR-*trnE*-*trnC*-*trnY*-*trnQ*-*trnI*-*trnA* between *srRNA* and *ND2*. The sister relationship between *T. remus* and other congeneric species is highly supported by phylogenetic analysis based on the protein-coding and ribosomal RNA gene sequences.

Family Scelionidae is a diverse group of parasitic wasps with more than 3,300 valid species worldwide (Samin and Asgari 2012). These wasps are generally tiny in size (0.5–12 mm). Most of them parasitize eggs of other insects, primarily those in order Orthoptera, Hemiptera, Neuroptera and Lepidoptera. Therefore, they can be promising biological control agents against agricultural pests (Galloway and Austin 1984; Austin 2005). *Telenomus remus* Nixon 1937 is a candidate parasitoid for control of some Lepidoptera pests, especially for the fall armyworm *Spodoptera frugiperda* (Kenis et al. 2019). In this study, the complete mitogenome of *T. remus* is determined and described.

The parental wasp samples were collected from Guangzhou city, Guangdong Province (23.09°N, 113.41°E). The voucher specimen (HYM002) was preserved in the Yunnan Agricultural University (Bin Chen, chbins@163.com). Total genomic DNA was extracted using the DNeasy Blood and tissue kit (Qiagen, Germany) following the manufacturer’s protocol. The complete mitogenome of *T. remus* was obtained by high-throughput sequencing on Illumina NovaSeq 6000 platform (San Diego, USA) with 150 bp paired-end reads and the average insert size was 350 bp in length. A total of 6 Gb clean data was obtained and used for assembly and assembled by IDBA-UD 1.1 (Peng et al. 2012) with minimum and maximum k values of 41 and 141 bp, respectively. The final mitogenome was identified through the Geneious 10.1.3 (http://www.geneious.com/), with an average sequencing depth of 956×. The preliminary annotation of the mitogenome was performed on MITOS (Bernt et al. 2013) to delimit gene boundaries and the result was further confirmed by alignment with homologous genes of the published *Telenomus* mitogenomes (KR270640, MF776884) using Geneious 10.1.3.

The complete mitogenome of *T. remus* is 16,014 bp in length, with 13 protein-coding genes (PCGs), 22 transfer RNA genes (tRNAs), two ribosomal RNA genes (rRNAs) and a control region (CR). The nucleotide composition is significantly biased toward adenine and thymine, with an A + T content of 85.4% (A = 44.9%, T = 40.5%, C = 8.5%, G = 6.1%), especially the control region, with the highest A + T content (95.7%), congruent with those of previously published parasitic wasps (Mao and Dowton, 2014). The mitogenome is skewed slightly toward A (0.052) and strongly toward C (0.164). The four most frequently used codons are all AT-rich codons, including TTA (Leu), ATT (Ile), TTT (Phe), and ATA (Met), which are used 471, 463, 430, and 322 times, respectively. In contrast, the C- and G-rich codons such as CGC (Arg), CCC (Pro) are rarely used. Similar bias in codon usage is also observed in other scelionid mitogenomes (Mao et al. 2012).

All PCGs use ATN as the start codon and stop with TAA or TAG. tRNAs were determined by the tRNAscan-SE (Lowe and Chan 2016) and ARWEN (Laslett and Canbäck 2008), and the secondary structures are typical cloverleaf structures except for the *trnS(AGN)* and *trnQ* with the dihydrouridine (DHU) arm formed a loop. The length of *lrRNA* and *srRNA* is 1,279 bp and 768 bp, respectively. Except for the CR (1,102 bp) located between *trnV* and *trnE*, there are 18 inter-genic regions, ranging from 1 to 74 bp, with the largest one located between *trnY* and *trnQ*. A total of 34 bp overlapped nucleotides between neighboring genes are found in eight locations, ranging from 1 to 17 bp in size.

When compared to the putative ancestral arrangement of insects and other egg parasitoids (Cameron 2014; Shen et al. 2019; Tang et al. 2019), *T. remus* mitogenome displays a novel gene order, with orders of 14 tRNAs are rearranged. This generated two novel gene clusters: the *trnN*-*trnF*-*trnS(AGN)*-*trnR* cluster between *ND3* and *ND5*, and the *trnM*-*trnV*-CR-*trnE*-*trnC*-*trnY*-*trnQ*-*trnI*-*trnA cluster* between *srRNA* and *ND2*. These two regions are also the hotspots of the gene rearrangements in the mitogenomes of Platygastroidea (Mao and Dowton 2014; Tang et al. 2019). It is interesting that the gene order is slightly difference in the three sampled *Telenomus* species. The *trnE* gene is positioned between CR and *trnC* in the mitogenome of *T. remus*, unlike *T*. sp and *T. dignus* where it is positioned between *trnN* and *trnF*.

Phylogenetic tree was constructed based on Bayesian inference of the dataset of the 13 PCGs and 2 rRNAs from 10 species in Platygastroidea and two outgroups (Figure 1), by using PhyloBayes MPI 1.7a (Lartillot 2013). The result supports the monophyly of the eight sampled species from Scelionidae, and the sister relationship between *T. remus* and other two *Telenomus* species is supported with high support value. Species identities of samples used in this study was further validated by DNA barcoding using *COI* fragment, which were 99.41% to 99.84% identical to the available sequences of *T. remus* from NCBI. The genetic distances between *T. remus* and two other *Telenomus* species (*T*. sp and *T. dignus*) were 15.2% and 11.4%, respectively.

**Figure 1.**
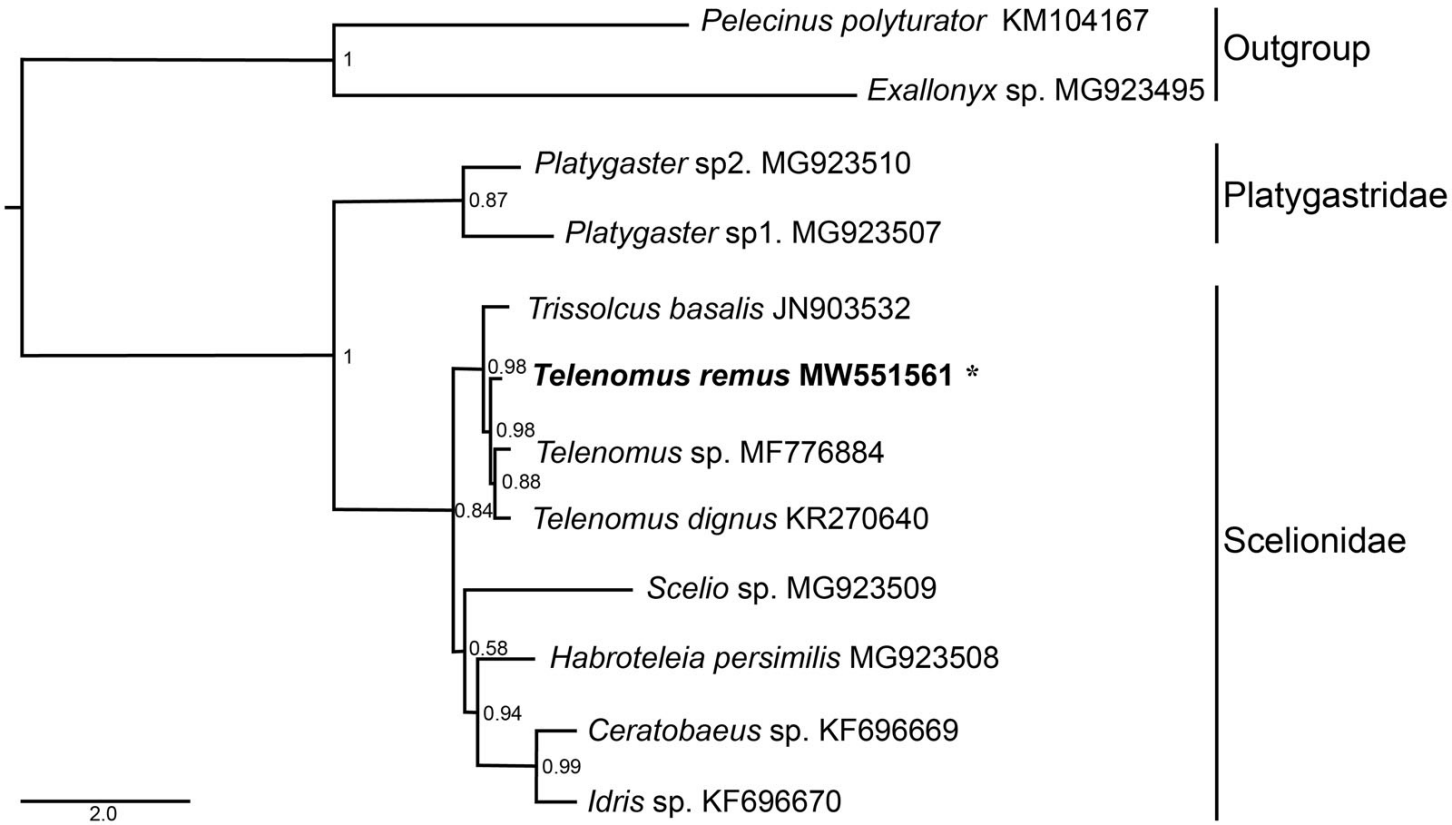
Phylogenetic relationship between *Telenomus remus* and other nine species from the superfamily Platygastroidea inferred from Bayesian inference of the 13 protein-coding genes and two rRNA genes. Supports at nodes are the Bayesian posterior probabilities. The newly sequenced mitogenome is highlighted by the asterisk.

## Data Availability

The genome sequence data that support the findings of this study are openly available in GenBank of NCBI at https://www.ncbi.nlm.nih.gov under the accession no. MW551561. The associated BioProject, SRA, and Bio-Sample numbers are PRJNA698262, SRR13589366 and SAMN17710193, respectively.
